# Sn-Decorated Cu/Cu_2_O Electrodes Enable Selective CO_2_ Reduction to Formic Acid under Realistic Flue Gas Streams

**DOI:** 10.1021/acsaem.5c03902

**Published:** 2026-01-28

**Authors:** Allef Leite, Eduardo Henrique Dias, Damilola Awotoye, Emmanuel Aransiola, Mohamed Ammar, Ernesto C. Pereira, Caue Ribeiro, Jonas Baltrusaitis

**Affiliations:** † National Nanotechnology Laboratory for Agribusiness (LNNA), Embrapa Instrumentation, São Carlos, São Paulo 13560-970, Brazil; ‡ Department of Chemistry-Physics, São Carlos Institute of Chemistry, University of São Paulo, São Carlos 13566-590, São Paulo, Brazil; § Department of Chemical and Biomolecular Engineering, 1687 Lehigh University, Bethlehem, Pennsylvania 18015, United States; ∥ Department of Chemistry, Federal University of São Carlos, São Carlos 13565-905, São Paulo, Brazil

**Keywords:** Cu/Cu_2_O/Sn electrocatalyst, CO_2_ electroreduction, formic acid (HCOOH), plasma-derived flue gas, oxide-derived copper

## Abstract

The electrochemical reduction of CO_2_ is typically investigated under pure CO_2_ feeds, but practical deployment must address more complex and dilute sources such as flue gases. Here, we studied Cu/Cu_2_O electrodes decorated with tin (Sn) synthesized using a scalable electrodeposition method and post-treatments under both pure CO_2_ and reactive nitrogen oxide-containing simulated flue gas, toward formic acid synthesis. Raman spectroscopy and Atomic Force Microscopy analyses revealed that flue gas exposure induces heterogeneous restructuring of the electrode with surface roughening, surface carbonate formation, and localized redeposition processes. Optimal catalyst performance under pure CO_2_ was achieved with intermediate Sn coverage of 3 min electrodeposition, delivering Faradaic efficiencies of 80% and production rates of 370 μmol cm^–2^ h^–1^. Sn-modified Cu_2_O electrodes also exhibited high selectivity toward formic acid under acidic gas containing simulated flue gas, reaching Faradaic efficiencies of 90% albeit at production rates of 113 μmol cm^–2^ h^–1^, despite a 10-fold reduced CO_2_ partial pressure. These results demonstrate that interfacial Sn–Cu structures enabled selective CO_2_RR even under challenging feed conditions, pointing out both the opportunities and limitations of translating laboratory-scale catalysts to realistic gas streams.

## Graphical abstract

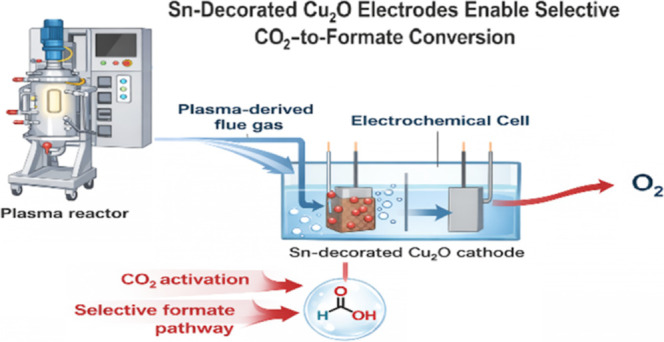

## 1 Introduction

The continuous rise in CO_2_ emissions from human activities and their associated environmental impacts has driven the development of renewable-powered technologies aimed at capturing CO_2_ and converting it into carbon-based fuels and chemicals. In recent years, the electrochemical reduction of carbon dioxide (CO_2_RR) into value-added products containing one or multiple carbon atoms (C_2_^+^) has emerged as a particularly promising approach.^1,2^ Both experimental and theoretical studies have played a crucial role in enhancing the activity and selectivity of CO_2_RR electrocatalysts.^3,4^ Strategies such as the synthesis of high-surface-area catalysts and the implementation of flow reactor designs to improve mass transport to the electrode surface have been employed to boost CO_2_ electroreduction performance.^5^

Most CO_2_RR studies rely on highly pure CO_2_ streams to minimize contaminants and avoid mass transport limitations. Capturing and purifying CO_2_ from power plant flue gas typically costs between $40 and $120 per ton,^6^ representing a significant portion of the final cost of electrochemical CO_2_RR products.^7^ For instance, technoeconomic analyses have shown that the cost of purified CO_2_ can account for approximately 15% and 30% of the operating expenses in the production of ethylene and carbon monoxide, respectively.^8^ Directly utilizing postcombustion flue gas as the CO_2_ feedstock can lower the overall cost of CO_2_ electrolysis by avoiding the capital and operational expenditures associated with CO_2_ capture, including absorption columns, heat exchangers, and stripping columns typically required for purification processes. However, flue gas from coal-fired power plants contains only about 6–18% CO_2_ by volume, diluted in nitrogen. This lower concentration reduces the CO_2_ flux and increases mass transport limitations during CO_2_RR, negatively impacting both current density and product Faradaic efficiency.^9^ The remaining composition primarily includes N_2_, O_2_, water vapor, and trace impurities such as NO_*x*_ or SO_*x*_.^10^ These conditions lead to low CO_2_ partial pressure,^11^ competitive side reactions,^12^ and complex surface chemistry on catalysts,^12^ making direct electroreduction far more challenging than in pure CO_2_ environments. Bridging this gap is essential to enable the large-scale implementation of electrochemical CO_2_ conversion technologies.

To investigate this, Luc et al.^13^ and Ko et al.^14^ explored the effects of NO (10,000 and 1000 ppm) and SO_2_ (8300 and 830 ppm), respectively, in CO_2_-containing feed streams. Their results showed a notable decline in product selectivity within a short period (30 min) at a current density of 100 mA cm^–2^. Additionally, Choi et al.^15^ evaluated the stability of SnS_*x*_ catalysts under exposure to 90 ppm of SO and NO_2_, observing consistent formate faradaic efficiency (FE) for 8 h at an optimized diluted CO_2_ concentration and 100 mA cm^–2^. Gautam and co-workers^16^ developed a dual methanol/water electrolysis configuration employing a Pb-based cathode, where the introduction of simulated flue gas containing about 50 ppm of SO_2_ and 50 ppm of NO caused only a minor change in product distribution. The main variation arose from oxygen dilution (a few percent O_2_), which affected partial current densities. Their results indicated a favorable formation of C_1_ oxygenated products. Even so, the impurity levels used were considerably lower than those typically found in industrial exhausts, and the study did not provide a detailed assessment of catalyst degradation or contaminant accumulation during extended operation. In another example, Van Daele et al.^12^ operated flow cells with flue-gas compositions representative of point-source emissions - approximately 13–14% CO_2_, 3–4% O_2_, and around 200 ppm each of SO_*x*_ and NO_*x*_. They achieved over 90% Faradaic efficiency for CO_2_ reduction to CO and formate, maintaining stable performance for roughly 20 h. However, the system was tested under relatively low current densities and primarily focused on gaseous reduction products, leaving the influence of acidic impurities on liquid-phase formate formation largely unexplored. Liu et al. demonstrated that an Ag_12_-bpy-NH_2_ metal–organic framework can adsorb CO_2_ from a 15% CO_2_ and 85% N_2_ mixture while maintaining high electrocatalytic performance. Under these dilute conditions, the catalyst achieved FE_CO_ = 96%.^17^ For formate formation, Li et al. reported low-coordination In­(III) single-atom sites anchored to a Zr-MOF monolayer, operating under strongly acidic conditions (pH 1.67). Their Zr-BTB-In catalyst delivered FE_HCOOH_ = 95–96% remained stable for 20 h. In a solid–electrolyte MEA configuration, it produced high-purity (100%) HCOOH solutions reaching 505.5 mmol L^–1^, representing one of the highest reported concentrations obtained directly in aqueous phase.^18^ More recently, Zhao et al. developed the conductive MOF Bi-HHTP, capable of capturing CO_2_ from a realistic flue gas like mixture (15% CO_2_/80% N_2_/5% O_2_) even at 40% relative humidity. The material retained strong CO_2_ affinity (*Q*_st_ approximately 45–49 kJ mol^–1^) and delivered nearly identical electroreduction performance in pure or dilute CO_2_ streams. In a solid–electrolyte MEA device, the system achieved FE_HCOOH_ = 91% under 15% CO_2_.^19^ Although these studies represent important progress in the electrochemical upgrading of dilute CO_2_, they still rely on complex MEA architectures, sophisticated crystalline frameworks (Ag-, In-, or Bibased MOFs).

Because the impact of acidic impurities on liquid-phase products remains poorly understood, researchers have turned their attention to materials capable of supporting multielectron CO_2_ reductions. Among these, oxide-derived copper electrodes have emerged as a particularly versatile platform.^20,21^ Their defect-rich surfaces, adjustable electronic structure, and large surface area promote the stabilization of critical reaction intermediates, thereby enabling multielectron reductions that yield both C_1_ and C_2_ products.^22,23^ Although oxide-derived copper exhibits high catalytic activity, its selectivity toward formic acid (HCOOH) remains limited because of competing hydrogen evolution and inadequate control over interfacial catalytic sites. Modifying the surface with secondary metals—particularly tin (Sn)—has been shown to enhance HCOOH selectivity. The formation of Sn–Cu interfacial sites helps stabilize the *OCHO intermediate, suppress hydrogen evolution, and promote more selective formate production^24,25^ but the specific effects of Sn coverage, the structural evolution of Sn–Cu interfaces, and their behavior under low-CO_2_, multicomponent gas feeds are still poorly understood. Previous studies suggest that catalyst performance depends not only on the initial surface composition but also on dynamic changes that occur during reaction.^26,27^ Under dilute CO_2_ conditions, such as those found in flue gas, processes like carbonate formation, local dissolution and redeposition, and surface roughening can strongly affect both activity and product selectivity.^16^

Building on these insights, we take an approach that directly investigates the dynamic behavior of Sn–Cu interfaces under realistic reaction conditions. We fabricate Sn-decorated Cu/Cu_2_O electrodes by electrodeposition followed by tailored thermal treatments to vary Sn distribution and alloying extent. Samples were characterized ex situ with complementary techniques (morphology: AFM; crystallography: XRD; surface chemistry: XPS and Raman; electrochemical: CV/LSV and chronoamperometry), while product analysis was performed with gas and liquid-phase techniques to quantify Faradaic efficiencies and formation rates. To assess the impact of realistic contaminants on catalyst activity, formate selectivity, and durability across reaction cycles, we benchmarked performance under both pure CO_2_ and plasma-derived simulated flue gas reactions. The simulated plasma flue gas was prepared from a gas cylinder and comprised approximately 9% CO_2_, 5% O_2_, 0.4% CO, 0.01% NO_*x*_, and 85% N_2_. This composition closely mirrors typical flue gas profiles from natural gas combustion, which generally contain around 8–10% CO_2_, 2–3% O_2_, and predominantly N_2_, with trace amounts of CO and NO_*x*_.^28^ Such alignment underscores the relevance and applicability of our experimental conditions to real-world scenarios correlating synthesis variables, surface speciation under reaction conditions, and electrochemical outcomes. We aim to identify concrete structure–performance relationships and to propose practical guidelines for designing Sn-modified Cu/Cu_2_O electrodes that combine selectivity with robustness in nonideal operating environments.

## 2 Experimental Section

### 2.1 Catalyst Synthesis

All solutions and glassware used were thoroughly prepared and cleaned using ultrapure deionized water (Millipore Milli-Q, 18.2 MΩ cm). Cu_2_O was electrochemically synthesized on a metallic copper substrate (99.995%) using a single-compartment three-electrode electrochemical cell, with a platinum wire as the counter electrode and an Ag/AgCl (3 M NaCl) electrode as the reference; all potentials reported herein are referenced against this electrode. The copper plates were polished and cleaned by ultrasonication in ethanol and water.

As described and adapted from previously reported procedures,^29^ CuSO_4_·5H_2_O (0.2 M, Sigma-Aldrich, ≥98%) was used as the Cu^2+^ source and 3 M lactic acid (Lab Alley, 90%) was added as the complexing agent at 55 °C and adjusted to pH 11.5 using NaOH (Sigma-Aldrich, 98%) and 5 M NaOH solution. Galvanostatic electrodeposition was performed by applying a potential of −0.42 V for 1 h using a potentiostat/galvanostat (Wave Driver 200, Pine Research). After synthesis, the film was rinsed with water, dried at room temperature, and stored for subsequent steps.

The electrode prepared in the previous step was decorated with Sn. For the Cu/Cu_2_O/Sn electrode, the same three-electrode setup was used, employing a 0.05 M SnCl_2_ solution (Sigma-Aldrich, ≥99.0%) and metallic tin (Sn^0^) was electrochemically deposited by applying a potential of −0.70 V for 1, 3, and 5 min. The samples were named Cu/Cu_2_O/Sn1, Cu/Cu_2_O/Sn3, and Cu/Cu_2_O/Sn5, respectively. After metal decoration, the electrodes were rinsed with water, dried at room temperature, and subsequently annealed in a furnace at 500 °C for 1 h.

### 2.2 Physical Characterizations of Cu/Cu_2_O/Sn Catalysts

X-ray diffraction (XRD) patterns of the synthesized materials were obtained using a PANalytical Empyrean X’Pert PRO diffractometer equipped with Cu Kα radiation (λ = 1.5418 Å). Data acquisition was carried out with the PANalytical software. The diffraction scans were collected over a 2θ range of 10.0° to 70.0°, employing a step size of 0.0131° and a counting time of 15.7 s per step, corresponding to a scan rate of 0.218838° s^–1^. The operating conditions of the X-ray source were set to 45 kV and 40 mA. A 4 mm mask was used during the measurements, together with a 1/16″ incident beam divergence slit and a 1/4″ incident beam antiscatter slit.

The Raman analyses were performed using a confocal microscope (WITec alpha300 R) with the laser operating with a 532 nm excitation source. A Zeiss LD Achroplan objective lens (20× magnification, NA 0.40, korr) was employed to focus the laser on the sample. Spectral acquisition covered the 0–4000 cm^–1^ range, centered at 2200 cm^–1^. The system utilized a diffraction grating with 600 lines per millimeter (G2:600 g mm^–1^), and the laser power at the sample surface was maintained around 5% of its maximum output. Each Raman spectrum was generated from three successive measurements, with each acquisition lasting 8 s. Prior to measurements, the instrument was calibrated against the characteristic silicon peak at 520 ± 1 cm^–1^.

Atomic Force Microscopy (AFM) was carried out on a Veeco 3100 Atomic Force Microscope, in tapping mode. All samples were imaged in air at a scan rate of 0.75 Hz with gold-coated silicon nitride (Si_3_N_4_) tips.

Surface chemical composition and elemental electronic states were examined using XPS on a SPECS system with a μ-FOCUS 600 monochromator under ultrahigh vacuum. Al Kα radiation (1486.7 eV, 100 W) was employed, and photoelectrons were analyzed with a PHOIBOS 1D-DLD hemispherical analyzer (0.85 eV resolution). Survey spectra were recorded at 100 eV pass energy, 1 eV step size, and 100 ms dwell time, while high-resolution spectra used 20 eV pass energy, 0.1 eV step, and 1 s dwell. Quantification relied on Scofield relative sensitivity factors,^30^ the instrument transmission function, and effective attenuation length corrections.^31^ The inelastically scattered background was subtracted using Shirley’s background.^32^ Data were processed and fitted in CasaXPS v2.3.6rev1.0Q,^33^ with no charge compensation applied.

Linear sweep voltammetry (LSV) was performed in the potential range from 0 to −1.6 V vs Ag/AgCl (3 M NaCl) at a scan rate of 5 mV s^–1^. The measurements were conducted under N_2_-saturated electrolyte prior to CO_2_ electroreduction, serving as a background reference, and repeated before and after CO_2_RR to evaluate possible changes in the electrode surface. Cyclic voltammetry (CV) was also recorded in the range of 0 to −1.6 V at a scan rate of 50 mV s^–1^ for 10 consecutive cycles, under N_2_ and CO_2_-saturated electrolyte. Electrode stability was further evaluated by chronoamperometry at −1.55 V, the potential at which the highest faradaic efficiency toward HCOOH formation was obtained, over 5 consecutive cycles of 1 h duration each.

### 2.3 Catalytic Performance

The CO_2_RR-to-HCOOH performance was evaluated for the Cu/Cu_2_O and Cu/Cu_2_O/Sn catalysts using an H-type electrochemical cell. The system employed 43 mL of 1 M KOH (Fisher Chemical, ≥85%) as the anolyte and 43 mL of 0.2 M KHCO_3_ (Lab Alley, 100%) saturated with CO_2_ (Airgas, 99.999%) as the catholyte, under ambient temperature conditions. A Ni foam (2.5 cm × 2.5 cm) was used as the counter electrode, separated from the working electrode by an anion exchange membrane (AEM, Fumasep FAS-30). An Ag/AgCl (3 M NaCl) electrode served as the reference. Before electrolysis, the catholyte was saturated with CO_2_ at a flow rate of 50 mL min^–1^ for 20 min, and during the CO_2_RR experiments, the flow rate was maintained at 10 mL min^–1^. KHCO_3_ is widely employed in CO_2_ electroreduction to formic acid because it provides a near-neutral, well-buffered environment that maintains high CO_2_ availability and favors selective HCOO^–^ formation over hydrogen evolution. A moderate concentration (0.2 M) ensures sufficient ionic conductivity without compromising CO_2_ solubility or transport.^34^ At the anode, Ni foam was selected as the counter electrode due to its high stability and robustness for the oxygen evolution reaction under alkaline conditions. Ni-based materials typically degrade or passivate under neutral media;^35^ therefore, alkaline KOH ensures stable OER performance and long-term structural integrity of the counter electrode. Using distinct electrolytes allows each half-cell to operate under optimized conditions: KHCO_3_ for selective CO_2_RR at the cathode and KOH for OER on Ni foam at the anode, while maintaining stable overall cell operation.

The same H-type electrochemical cell was employed for CO_2_RR supplied with the outlet stream (plasma-derived flue gas) generated by a dielectric-barrier discharge (DBD) reactor. In this setup, already described by Gonzalez-Casamachin et al.,^36^ 200 mg of catalyst were packed into the discharge region and held in place with quartz wool. The reactor was constructed using a stainless-steel inner electrode (O.D. = 16 mm) inserted into a borosilicate glass tube (I.D. = 20 mm), which was externally wrapped with copper tape serving as the outer electrode over an active discharge length of 140 mm. A heating jacket surrounding the outer electrode maintained the reactor wall temperature above 140 °C. Feed gases (CO_2_, O_2_, and N_2_) were supplied from compressed cylinders and metered by a Brooks mass flow controller. Water vapor was added by saturating the gas stream before entering the reactor, and all lines were heated to keep the mixture above 110 °C to prevent condensation. Once the targeted flow rates and vapor conditions were stabilized, plasma was ignited at a defined discharge power. The outlet composition was monitored every 10 min, and steady-state operation was assumed after three consecutive measurements with no further changes. This plasma-derived flue gas was continuously delivered to the catholyte of the H-cell for subsequent CO_2_RR experiments at a rate of 10 mL min^–1^. The simulated flue gas used in this study was prepared from a gas cylinder and consisted of approximately 9.555% CO_2_, 5% O_2_, 0.445% CO, 0.010% NO_*x*_, and 84.99% N_2_. Expressed in parts per million, this corresponds to 95,550 ppm of CO_2_, 50,000 ppm of O_2_, 4450 ppm of CO, 100 ppm of NO_*x*_, and 849,900 ppm of N_2_.

#### 2.3.1 Product Quantification and Faradaic Efficiency Calculations

High-performance liquid chromatography (HPLC) was employed to detect and quantify formic acid (HCOOH) using an Agilent 1260 Infinity system equipped with a multiwavelength detector (MWD) set to 210 nm. Separation was performed on a Hi-Plex H column (300 mm × 7.7 mm, Agilent) maintained at 45 °C. The mobile phase consisted of 0.2 M H_3_PO_4_ in ultrapure water, delivered at a constant flow rate of 1.0 mL min^–1^. A sample injection volume of 10 μL was used, with a total run time of 10 min. The retention time of HCOOH was identified at 3.35 min. Quantification was carried out using a five-point external calibration curve (1, 3, 10, 30, and 100 mM), with a linear regression coefficient (*R* ^2^) of 0.999. Peak area integration was normalized using acetic acid as an internal standard, and a response factor was applied to determine the final HCOOH concentration.

The total number of moles of liquid product (*n*_i_), where i is the product, was obtained by multiplying the concentration by the electrolyte volume (*V*_el_)­

1 $$n_{i}=C_{i}\times V_{el}$$

The Faradaic efficiency (FE) of HCOOH was calculated according to

2 $${FE}_{HCOOH}=\frac{Z_{HCOOH}\times F{\times n}_{HCOOH}}{Q}\times 100\%$$

where *Z*_HCOOH_ being the number of electrons required to form one molecule of HCOOH and *Q* is the total charge passed during electrolysis, determined by integrating the current over time.

The gaseous products were analyzed by gas chromatography (GC, SRI 8610C Multi-Gas no. 5 Configuration) equipped with a thermal conductivity detector (TCD). Calibration curves for H_2_ and CO were constructed from standard gas mixtures and yielded concentrations directly in μmol L^–1^. During electrolysis, the outlet gas stream was maintained at a constant flow of 10 mL min^–1^, from which 10 mL aliquots were injected into the GC. The GC calibration allowed the direct determination of the analyte concentration (*C*_i_), where i refers to H_2_ or CO, in the injected volume. The molar production rate of each gaseous product was calculated as

3 $${ṅ}_{i}=C_{i}\times Q_{out}$$

where *Q*_out_ is the outlet flow rate (0.010 L min^–1^). The total number of moles formed during electrolysis was obtained by integrating the molar production rate over the reaction time

4 $$n_{i}={ṅ}_{i}\times t$$

where *t* is the total electrolysis time. Faradaic efficiencies (FE) were then calculated according to eq 2.

## 3 Results and Discussion

### 3.1 Physical Characterizations of Cu/Cu_2_O and Cu/Cu_2_O/Sn Catalysts

The XRD and Raman data (Figure 1) illustrate the structural evolution of the as-synthesized Cu/Cu_2_O films before and after annealing, as well as following the CO_2_RR. In the diffractograms (Figure 1a), the main features corresponded to Cu_2_O, with well-defined peaks observed at 29.2°, 36.1°, 41.9°, and 61.1° were indexed to the (110), (111), (200), and (220) planes, respectively indexed according to JCPDS 03-0898,^37^ as also observed by Lam et al. (2022).^38^ For the annealed samples, XRD did not reveal any distinct new phase; instead, an additional peak appeared (41.9°), which could also be attributed to Cu_2_O. This extra reflection likely corresponded to a less intense Cu_2_O plane, reflecting increased crystallinity rather than the formation of a new phase. These observations indicated that no bulk crystalline compound other than Cu_2_O was formed, and no evidence of crystalline SnO_2_ or CuO was detected by XRD.

**Figure 1:**
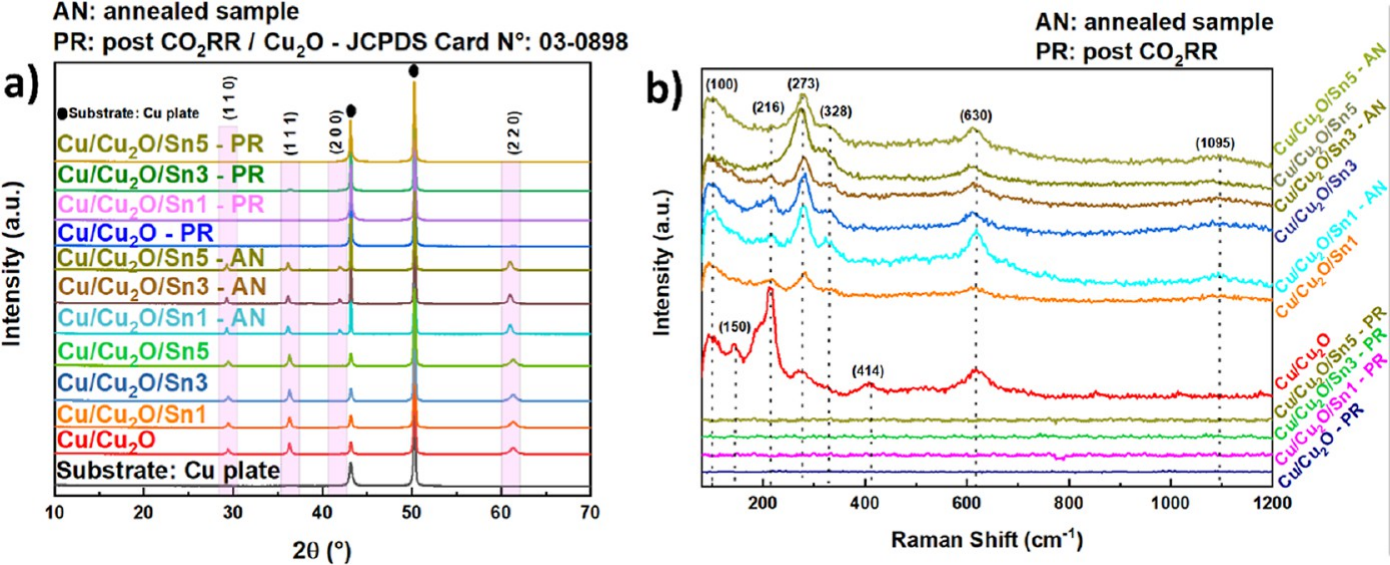
(a) XRD patterns and (b) Raman spectra of Cu/Cu_2_O and Cu/Cu_2_O/Sn composites prepared by Sn deposition for 1, 3, and 5 min, followed by annealing (AN) and postreaction (PR) analysis.

The Raman spectra (Figure 1b) revealed bands at 273 and 328 cm^–1^, which could be attributed to CuO.^39^ The absence of corresponding CuO reflections in XRD was likely due to the small quantity, high dispersion, or amorphous nature of the CuO phase, which rendered it undetectable by XRD. Thus, Raman provided complementary information, capturing subtle surface or near-surface oxidation to CuO. The characteristic bands observed in the Raman spectra are listed in Table 1. The peak at 100 cm^–1^ is related to intrinsic Cu_2_O lattice vibrations^40^ that are defect-activated, indicating the presence of structural imperfections in the lattice. Similarly, the 150 cm^–1^ peak corresponds to acoustic phonons of Cu_2_O^41^ that are also activated by defects, reflecting the sensitivity of the lattice dynamics to local disorder. The 216 cm^–1^ band arises from a second-order lattice vibration of Cu_2_O.^42^ In addition to these Cu_2_O features, peaks at 273 cm^–1^ and 328 cm^–1^ can be assigned to the A_g_ and B_g_ modes of CuO,^39^ respectively, as mentioned earlier. These bands signal the formation of a thin CuO layer on the surface of Cu_2_O, likely due to partial oxidation during synthesis and annealing. Finally, the 414 cm^–1^ peak is characteristic of an intrinsic phonon of Cu_2_O,^43^ reinforcing the presence of the main Cu_2_O phase.

**Table 1:** Raman Band Assignments of Cu/Cu_2_O and Cu/Cu_2_O/Sn Composites, with Comparison to Literature Values

Raman shift (cm^–1^)	Associated phase/compound	Literature value (cm^–1^)	Reference
100	Intrinsic Cu_2_O lattice (defect-activated)	∼ 110	^40^
150	Cu_2_O (acoustic phonons activated by defects)	∼ 150	^41^
216	Cu_2_O (second-order lattice vibration)	∼ 218	^42^
273	CuO – A_g mode	∼270	^39^
328	CuO – B_g mode	∼321	^39^
414	Cu_2_O intrinsic phonon	∼ 415	^43^
630	Cu_2_O (IR mode made Raman-active)	∼ 625	^39^
1095	Surface or solution carbonate	∼ 1085–1095	^44^

After CO_2_RR, clear changes were observed in both Raman and XRD analyses. All the characteristic Cu_2_O Raman bands (100, 150, 216, 273, 328, 414, and 630 cm^–1^) disappeared, showing that the oxidized Cu_2_O phase was no longer present. In the XRD patterns, only the peaks from the Cu substrate remained, with two strong reflections at 43.2° and 74.0°, corresponding to the (111) and (200) planes of metallic Cu (JCPDS 04-0836).^45^ A residual Cu_2_O diffraction signal assigned to the (111) plane was detected exclusively for the Cu/Cu_2_O/Sn3 electrode (Figure 1a) postreaction, while it was absent in the Sn free, Cu/Cu_2_O/Sn1, and Cu/Cu_2_O/Sn5 electrodes. This selective retention arises from an optimal, intermediate Sn deposition time, which stabilizes the intrinsically low surface energy Cu_2_O­(111) facet through Cu–Cu_2_O–Sn interfacial interactions. At lower Sn coverage (1 min), the interfacial interaction is insufficient to prevent complete Cu_2_O reduction, whereas excessive Sn deposition (5 min) promotes over reduction and surface segregation, eliminating residual Cu_2_O altogether. These results indicate that the Cu_2_O was largely—or even completely—reduced to metallic Cu during the reaction, alongside noticeable surface restructuring. Overall, the combined XRD and Raman analyses demonstrated that Cu_2_O was the predominant phase before the reaction, reorganized upon annealing, and was subsequently electrochemically reduced, while Sn remained structurally undetectable, likely influencing only surface chemistry or oxide stability. Thus, Sn likely acted by modulating the Cu surface chemistry and stabilizing interfacial sites, rather than forming a distinct crystalline phase.^46^

Figure 2a–c shows linear sweep voltammetry (LSV) curves for Cu/Cu_2_O/Sn electrodes, measured before and after the CO_2_RR, and compared with a blank under N_2_ atmosphere. For all electrodes, the polarization plots saturated by N_2_ (blue dashed line) exhibited relatively low and symmetric currents, indicating the absence of significant redox processes within the studied potential range. Prior to CO_2_RR (dark-colored lines), all electrodes displayed a decrease in current density at negative potentials, suggesting initial electrochemical activity associated with the Cu/Cu_2_O/Sn surface. A subtle fluctuation observed at approximately −0.8 V in the Cu/Cu_2_O/Sn5 LSV curve (Figure 2c) appears only in the Sn rich sample and is reproducible across independent measurements. Similar small prewaves have been reported for Cu/Sn materials and are commonly attributed to potential-dependent adsorption of CO_2_RR intermediates (CO*, OCHO*) or to minor redox transitions involving Sn containing surface species during the early stages of oxide derived catalyst activation.^47^ Since all electrodes were tested under identical hydrodynamic conditions, and this feature is absent in Cu/Cu_2_O/Sn1 and Cu/Cu_2_O/Sn3, a mass-transport origin is unlikely. Therefore, this deviation is most plausibly associated with a surface-limited quasi-redox/adsorption process specific to the higher Sn loading in Cu/Cu_2_O/Sn5. N_2_-saturated measurements showed only baseline currents, confirming that the observed potential increase under CO_2_ was associated with CO_2_RR. For Cu/Cu_2_O/Sn1 (Figure 2a), a slight increase at more negative potentials indicated partial electrode activation. Cu/Cu_2_O/Sn3 (Figure 2b) showed a more pronounced increase, especially below −1.2 V, suggesting that intermediate Sn deposition times promoted a more active CO_2_RR surface.^48^ In contrast, Cu/Cu_2_O/Sn5 (Figure 2c) exhibited only minor activation, consistent with a less favorable surface state, probably as a result of oversaturation of the electrode surface with Sn. As suggested by the LSV curve, the Cu/Cu_2_O/Sn3 sample appeared to be the most active for CO_2_ reduction and was therefore selected for XPS analysis at all stages of synthesis and after the reaction. Tafel slope analysis (Figure 2d) was conducted to evaluate how Sn loading modulates the charge transfer kinetics of the Cu/Cu_2_O catalyst. The Cu/Cu_2_O/Sn3 sample exhibited the smallest Tafel slope (73 mV dec^–1^), followed by Cu/Cu_2_O/Sn1 (97 mV dec^–1^), while Cu/Cu_2_O/Sn5 showed a higher value (198 mV dec^–1^). This trend clearly indicates that moderate Sn decoration (3 min) accelerates the rate of the potential determining electron transfer step, whereas excessive Sn coverage (5 min) introduces kinetic limitations, likely due to partial blocking of the Cu_2_O/Sn interfacial sites responsible for stabilizing key CO_2_RR intermediates. The superior kinetic behavior of Cu/Cu_2_O/Sn3 correlates directly with its highest HCOOH formation rates and Faradaic efficiencies among the others catalysts. In contrast, the steep slope of Cu/Cu_2_O/Sn5 implies slower interfacial charge transfer and a shift toward less favorable reaction energetics, aligning with its diminished CO_2_RR performance. These results highlight that controlling Sn loading is essential for tuning the reaction kinetics and maximizing formate selectivity. Electrochemical impedance spectroscopy (EIS) was performed to evaluate how Sn decoration influences interfacial charge-transfer kinetics on the Cu/Cu_2_O surface.

**Figure 2:**
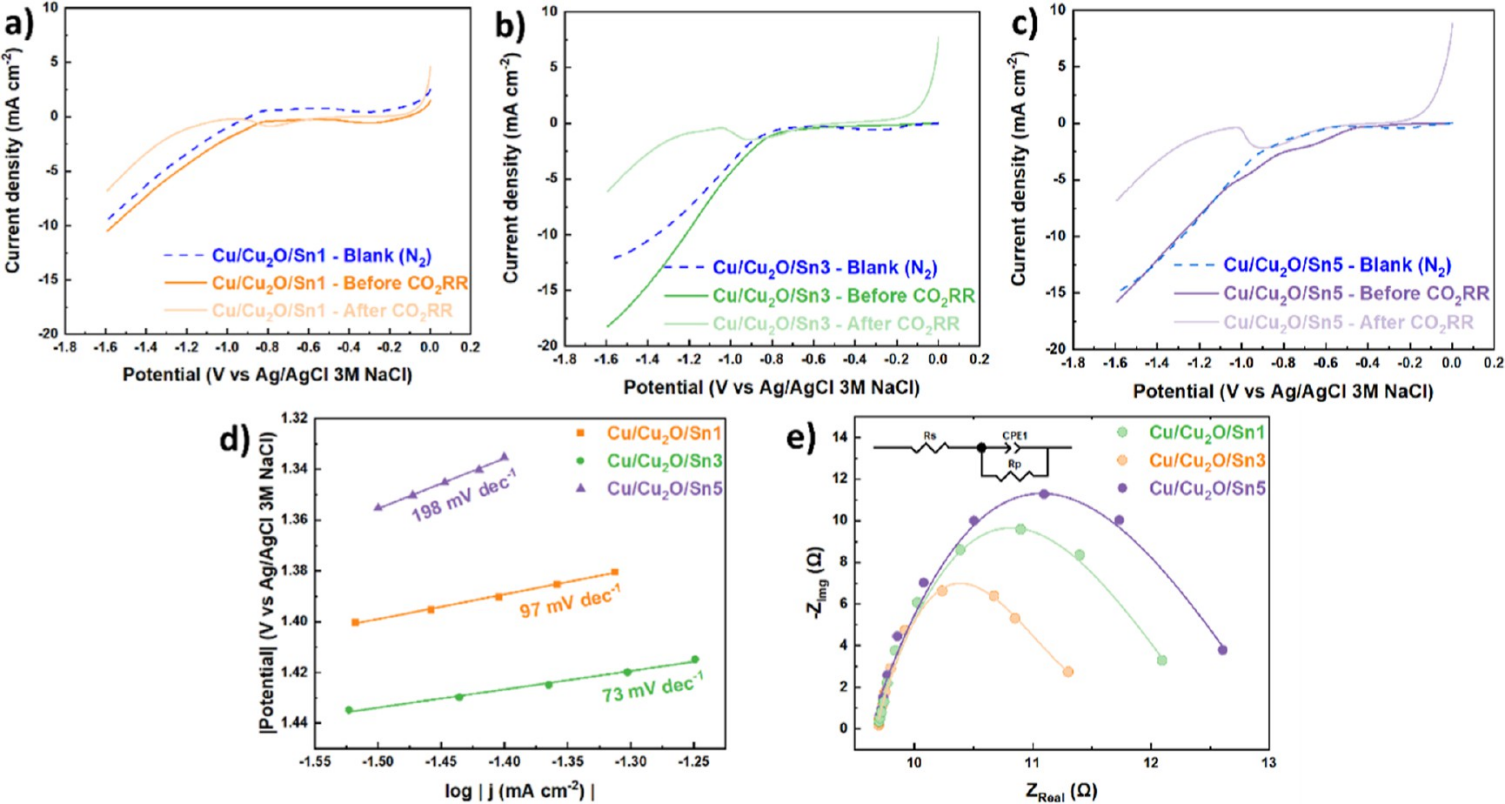
Linear sweep voltammetry (LSV, (a–c), scan rate 5 mV s^–1^) of (a) Cu/Cu_2_O/Sn1, (b) Cu/Cu_2_O/Sn3, (c) Cu/Cu_2_O/Sn5 samples, (d) Tafel plots derived from the corresponding LSV curves and (e) Nyquist plots and fitted curves obtained from electrochemical impedance spectroscopy (EIS) measurements for the same samples. All experiments were carried out in a two-compartment electrochemical cell using 0.2 M KHCO_3_ as the catholyte and 1 M KOH as the anolyte.

The Nyquist fits (Figure 2e and Table 2) revealed that all samples exhibit similar solution resistance (*R*_s_ approximately 9.71 Ω), indicating that variations in performance originate exclusively from differences in interfacial processes rather than changes in electrolyte conductivity. In contrast, the charge-transfer resistance (*R*_p_) varied significantly with Sn loading: Cu/Cu_2_O/Sn3 displayed the lowest *R*_p_ (1.637 Ω), while Cu/Cu_2_O/Sn1 and Cu/Cu_2_O/Sn5 exhibited higher values of 2.867 Ω and 2.393 Ω, respectively. The minimized *R*_p_ for Sn3 confirms that moderate Sn coverage optimizes the electronic environment at the Cu_2_O/Sn interface, enabling more efficient electron transfer during CO_2_ activation. The CPE parameters support this interpretation. Cu/Cu_2_O/Sn3 showed the highest CPE-T value (0.004), consistent with increased interfacial capacitance and a larger electrochemically accessible surface. The χ^2^ values (10^–3^ range across all samples) confirm high-quality fits. These EIS trends mirror both the Tafel behavior and CO_2_RR performance. The sample with the lowest charge-transfer resistance (Cu/Cu_2_O/Sn3) is the same that delivers the highest HCOOH formation rate and Faradaic efficiency, indicating that rapid electron transfer plays a central mechanistic role on Cu_2_O/Sn interfaces. In contrast, the higher *R*_p_ values of Cu/Cu_2_O/Sn1 and Cu/Cu_2_O/Sn5 correlate with their inferior kinetics and lower catalytic output, demonstrating that insufficient (1 min) or excessive (5 min) Sn coverage disrupts the optimal balance between electronic modulation and available active sites. Overall, the EIS data confirm that the Cu/Cu_2_O/Sn3 configuration provides the most favorable kinetic environment for CO_2_ electroreduction.

**Table 2:** EIS Parameters for Cu/Cu_2_O/Sn1, Cu/Cu_2_O/Sn3 and Cu/Cu_2_O/Sn5 Electrodes

Catalyst	Element	Value
Cu/Cu_2_O/Sn1	*R*_s_	9.715 Ω
	*R*_p_	2.867 Ω
	CPE-T	0.003
	χ^2^	3 × 10^–3^
Cu/Cu_2_O/Sn3	*R*_s_	9.713 Ω
	*R*_p_	1.637 Ω
	CPE-T	0.004
	χ^2^	1 × 10^–3^
Cu/Cu_2_O/Sn5	*R*_s_	9.712 Ω
	*R*_p_	2.393 Ω
	CPE-T	0.003
	χ^2^	2 × 10^–3^

The XPS results (Figure 3) show how the surface composition and oxidation states of the Cu/Cu_2_O/Sn3 samples evolve throughout the different preparation steps. In the survey spectra (Figure 3a), the main signals correspond to Cu, O, and Sn, confirming the successful deposition of tin on the Cu/Cu_2_O. The weak C 1s signal mainly originates from surface carbon contamination, which is expected for air-exposed samples. The Cu/Cu_2_O sample exhibits a Cu 2p_3/2_ main peak centered at approximately 932.4 eV, which is characteristic of reduced copper species (Cu^0^/Cu^+^), while the presence of satellite features between 941 and 944 eV indicates the coexistence of Cu^2+^ species at the surface.^49,50^ Due to the well-known overlap of binding energies for Cu^0^ and Cu^+^ in the Cu 2p region, these species cannot be unambiguously distinguished based solely on Cu 2p spectra. After annealing, pronounced changes are observed in the Cu 2p region. The intensity of the main peak at 932.4 eV decreases, while the contribution associated with Cu^2+^ at 933.8 eV becomes more prominent. Concurrently, the Cu^2+^ satellite features between 941 and 944 eV increase in intensity, indicating enhanced surface oxidation. These observations suggest partial oxidation of reduced copper species during annealing, likely promoted by residual oxygen, resulting in a surface composed of both Cu_2_O related reduced copper species and CuO. Sn^0^ component was present only in the as deposited Cu/Cu_2_O/Sn3 sample. Upon annealing, Sn is fully oxidized to a Sn^4+^ oxide (SnO_2_), as confirmed by XPS, so that the electrodes used for CO_2_RR begin with a purely Sn^4+^ surface. Postreaction XPS (after CO_2_RR) likewise shows only Sn^4+^, with no detectable metallic Sn, indicating that the Sn remains oxidized under CO_2_RR conditions, i.e., no electrochemical reduction of SnO_2_ to Sn^0^ occurred during operation. The O 1s spectra (Figure 3d) show a component around 530.5 eV attributed to lattice oxygen and a secondary feature at 532 eV related to surface hydroxyls or adsorbed oxygen species.^51^ The XPS survey spectra were analyzed to determine the surface composition of the samples, the Cu/Cu_2_O sample exhibited a high oxygen content (81.9 at %) and a copper fraction (17.9 at %). After Sn deposition, the Cu_2_O/Sn3 sample exhibited a slight decrease in copper (16.3 at %) and the presence of tin at 17.8 at %, while oxygen remained nearly at 65.9 at %, indicating the successful deposition of tin on the surface. Following the annealing step, the same sample showed a further decrease in oxygen content to 66.1 at %, a slight decrease in copper (13.9 at %), and an increase in tin (20.0 at %), consistent with surface restructuring and the formation of a Cu–Sn interface. Overall, these results demonstrate that the deposition and subsequent annealing effectively modified the surface composition of Cu_2_O, introducing tin while adjusting the relative amounts of copper and oxygen.

**Figure 3:**
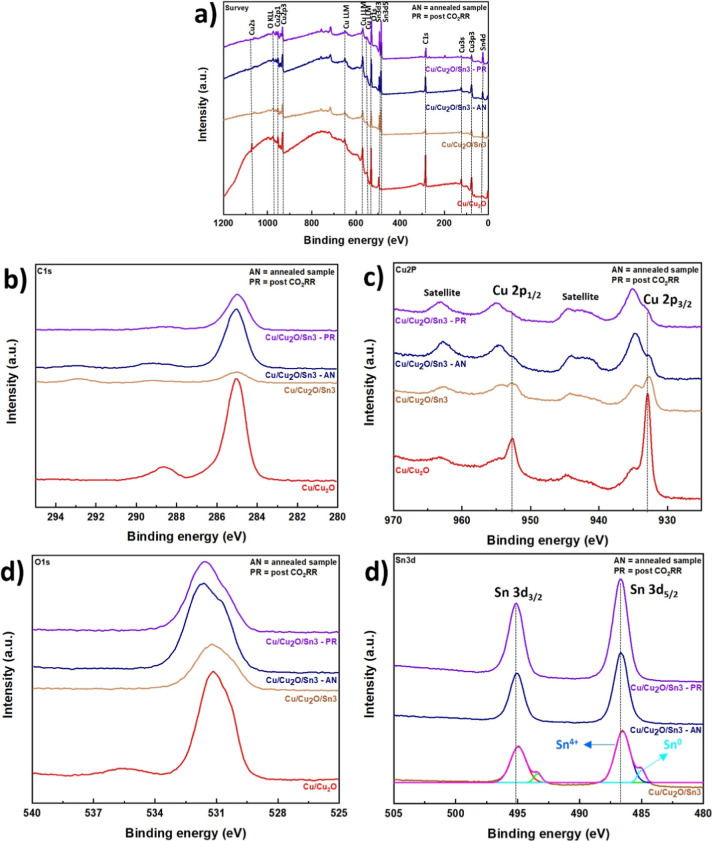
XPS spectra at different stages: Cu/Cu_2_O, Cu/Cu_2_O/Sn3 after Sn deposition, annealed (Cu/Cu_2_O/Sn3-AN), and after CO_2_RR (Cu/Cu_2_O/Sn3-PR). (a) Survey spectra showing the main elements present in the samples. (b) High-resolution C 1s spectra. (c) Cu 2p spectra. (d) O 1s spectra. (e) Sn 3d spectra.

The AFM images presented in Figure 4 provided insights into the morphological evolution of Cu/Cu_2_O decorated with Sn for different deposition times (1, 3, and 5 min), as well as the corresponding surfaces after annealing at 500 °C for 1 h.

**Figure 4:**
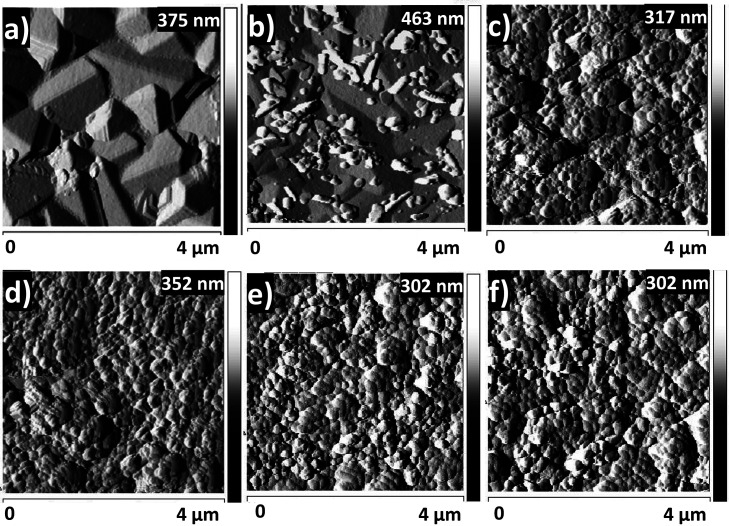
AFM images of Cu/Cu_2_O films decorated with Sn at different deposition times: (a) 1 min, (b) 3 min, and (c) 5 min. The corresponding samples after annealing at 500 °C for 1 h are shown in (d) 1 min, (e) 3 min, and (f) 5 min.

For the as-deposited samples (Figure 4a–c), the effect of Sn decoration time was evident. After 1 min of deposition (Figure 4a), the surface exhibited a homogeneous surface, with only isolated points of Sn and Cu_2_O facets still visible. At 3 min (Figure 4b), surface coverage increased, resulting in a more continuous granular texture. At 5 min (Figure 4c), the Sn layer almost entirely covered the Cu_2_O surface, forming a more compact coating, although the granular morphology remained irregular. This evolution is consistent with the progressive increase in Sn loading. After the thermal treatment (Figure 4d–f), clear morphological changes were observed. The granular features became more compact, with evidence of particle coalescence and surface densification. These modifications were consistent with previous literature reports.^52,53^ Annealing of Cu_2_O and related copper oxide films typically induced grain growth, rounding of sharp edges, and increased surface roughness due to thermally driven diffusion and coalescence. The evolution observed in the present samples suggested that annealing promoted restructuring of the Sn-decorated Cu_2_O surface, resulting in more interconnected features compared to the as-deposited state. In addition to these morphological changes, annealing was also expected to strongly influence the chemical and structural properties of the catalyst. Thermal treatment at 500 °C could promote the partial reduction of Cu_2_O, facilitate Sn diffusion into the Cu matrix, and lead to the formation of Cu–Sn intermetallic phases.^54,55^

Figure 5a–c presented cyclic voltammograms (CVs) of Sn-decorated Cu/Cu_2_O electrodes, multiple redox currents arose from the reduction and oxidation of Cu and Sn species, as well as from electrochemically induced structural transformations. At more negative potentials (approximately below −0.6 V), a sharp increase in cathodic current was observed, attributable to the reduction of Cu_2_O to metallic Cu (Cu^0^). Such behavior has been well documented for oxide-derived Cu electrodes, which often undergo partial reconversion to metallic Cu under negative polarization conditions.^56,57^ Measurements were performed under N_2_ (dashed blue line) and CO_2_ (solid line) atmospheres, enabling comparison between background currents and the electrochemical response during CO_2_ reduction. The moderate hysteresis between forward and reverse scans suggested partially reversible processes with some surface restructuring during polarization. The CV profile indicated enhanced redox contributions, consistent with the formation of additional active sites that stabilized intermediates involved in CO_2_ reduction. Cu/Cu_2_O/Sn5 (Figure 5c) exhibited a broader hysteresis, implying more extensive surface modifications, likely due to dynamic restructuring of Sn species during the reaction, and the higher Sn loading, which appeared to partially suppress CO_2_ reduction activity. Notably, the cathodic peak observed under N_2_ was higher than that under CO_2_, suggesting that excessive Sn coverage may have blocked Cu active sites or altered the electronic properties of the surface, leading to a higher contribution from background capacitive or Sn-related reduction processes rather than selective CO_2_RR. Such effects were consistent with the generation of additional active sites and the possible formation of metallic or intermetallic phases during the CV scans themselves.^58,59^ Additionally, thermal treatment (annealing) at 500 °C promoted Sn diffusion into the Cu matrix, partial reduction of Cu_2_O, and the formation of Cu–Sn intermetallic phases (e.g., Cu_3_Sn or Cu_6_Sn_5_), which may have persisted during electrocatalysis.^60^ The presence of these intermetallics could have modified the electronic density of states and adsorption potentials for reaction intermediates, contributing to the observed selectivity toward HCOOH formation.^61,62^ Overall, the CV data confirmed that Sn incorporation enhanced the electrocatalytic response toward CO_2_ reduction, with performance strongly dependent on deposition time.

**Figure 5:**
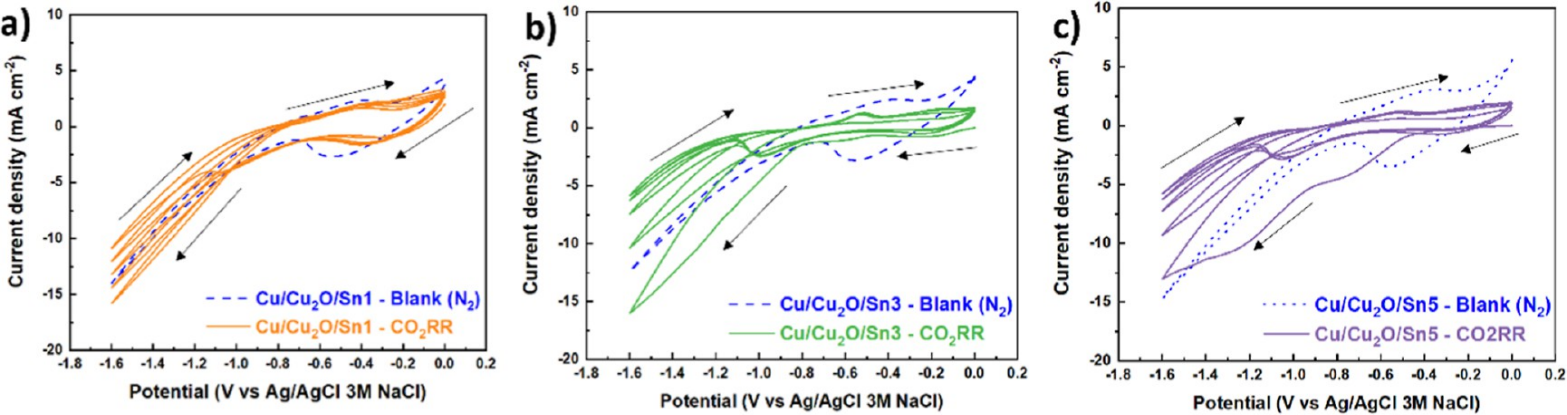
Cyclic voltammetry (CV, (a–c) scan rate 50 mV s^–1^) of (a) Cu/Cu_2_O/Sn1, (b) Cu/Cu_2_O/Sn3 and (c) Cu/Cu_2_O/Sn5. Measurements were performed before CO_2_RR and compared with a blank electrode under N_2_ atmosphere (blue dashed line). All experiments were carried out in a two-compartment electrochemical cell using 0.2 M KHCO_3_ as the catholyte and 1 M KOH as the anolyte.

### 3.2 CO_2_RR Performance

Chronoamperometric measurements (Figure 6) were performed at different applied potentials to evaluate the CO_2_RR behavior of Cu/Cu_2_O/Sn electrodes. The current density initially exhibited a sharp transient peak, followed by a gradual stabilization into a quasi-steady-state regime. This behavior was attributed to a combination of capacitive charging of the electrode/electrolyte interface and possible surface transformations, such as the partial reduction of surface oxides, before reaching a dynamic steady state during CO_2_ electroreduction.^20,63^ Fluctuations observed in some of the curves were likely associated with gas bubble formation and detachment, or with local variations in mass transport at the electrode surface.

**Figure 6:**
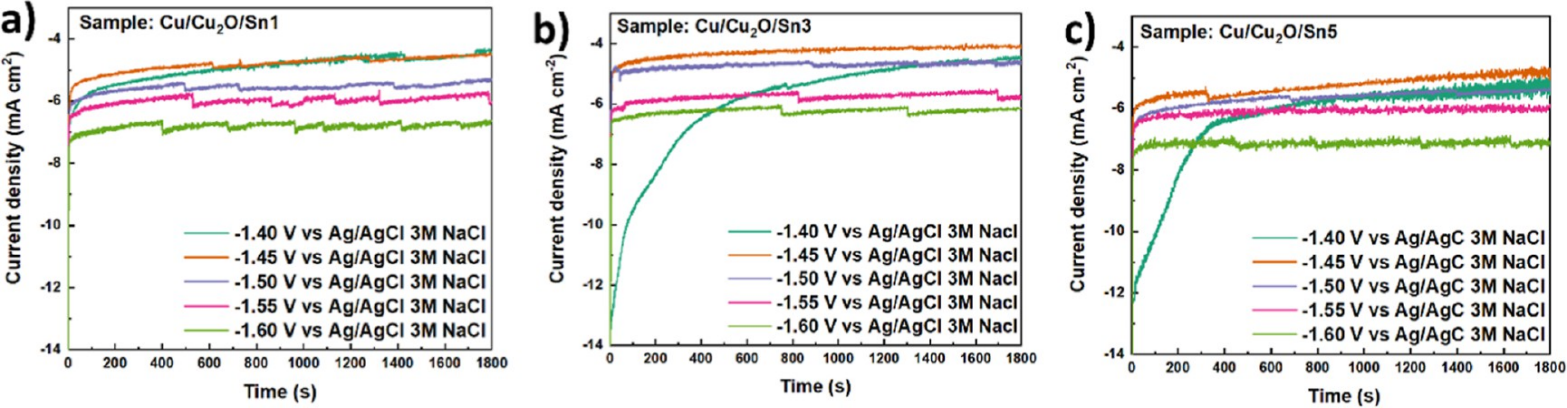
Chronoamperometric curves of Cu/Cu_2_O/Sn electrodes prepared with different Sn deposition times (a) 1, (b) 3 and (c) 5 min at various applied potentials (−1.40 to −1.60 V vs Ag/AgCl 3 M NaCl).

The electrode prepared with 1 min of Sn deposition (Cu/Cu_2_O/Sn1) displayed the lowest current densities across the entire potential range. This suggested that the limited amount of Sn introduced at this deposition time did not substantially modify the catalytic surface of Cu_2_O, resulting in reduced overall activity for CO_2_ reduction. The relatively stable but low current densities indicated that the catalytic surface area or the density of Sn-related active sites was insufficient to enhance the desired reaction pathways. In contrast, the Cu/Cu_2_O/Sn3 electrode showed the most favorable performance. This sample exhibited slightly higher steady-state current densities compared to Cu/Cu_2_O/Sn1, together with the highest Faradaic efficiency (FE) toward HCOOH among all tested catalysts. The correlation between increased activity and improved selectivity indicated that the deposition time of 3 min provided an optimal Sn coverage, leading to the formation of Sn–Cu interfacial sites, which are known to promote the formation of formate intermediates while suppressing competing hydrogen evolution.^61^ At −1.40 V, the Cu/Cu_2_O/Sn3 and Cu/Cu_2_O/Sn5 electrodes exhibit a prolonged current stabilization period of approximately 1000 and 800 s respectively, which is attributed to a slow electrochemical activation process rather than experimental artifacts. This behavior was reproducible across triplicate measurements and was not observed at more negative potentials or for the Cu/Cu_2_O/Sn1 electrode. The delayed stabilization at low overpotential is associated with gradual surface restructuring, including the reduction of SnO_*x*_ species, evolution of Cu–Sn interfacial sites, and the establishment of steady-state adsorption of CO_2_ reduction intermediates. Similar activation-controlled current transients have been reported for Cu-based and oxide-derived catalysts operating at low overpotentials. After stabilization, the current reaches a steady-state regime representative of the intrinsic catalytic performance.^64–66^ The stabilization of the current over time further suggested that the catalytic surface remained active under the applied conditions without significant deactivation within the investigated time window. For the Cu/Cu_2_O/Sn5 electrode, the initial current densities were comparable to or even higher than those of Cu/Cu_2_O/Sn3. However, the steady-state currents tended to be less stable, and the FE for HCOOH was lower. This behavior may have been related to the excessive Sn loading, which could have formed a thicker overlayer that masked the underlying Cu_2_O surface, altered the balance of active sites, or introduced mass transport limitations. As a consequence, despite the higher initial activity, the overall selectivity toward HCOOH was reduced.

The electrochemical reduction of CO_2_ to HCOOH was initially evaluated using Cu/Cu_2_O without Sn deposition, to isolate the effects associated with Sn surface decoration (Figure 7a). Cu/Cu_2_O exhibited measurable catalytic activity, with the HCOOH formation rate increasing steadily from −1.40 to −1.60 V. However, the Faradaic efficiency remained below 55% across the entire potential range, indicating that a substantial fraction of the current was consumed by competing side reactions. Control experiments confirmed that neither metallic Cu (substrate) nor the blank experiment under N_2_ purging produced detectable amounts of HCOOH, highlighting the intrinsic catalytic role of Cu_2_O.

**Figure 7:**
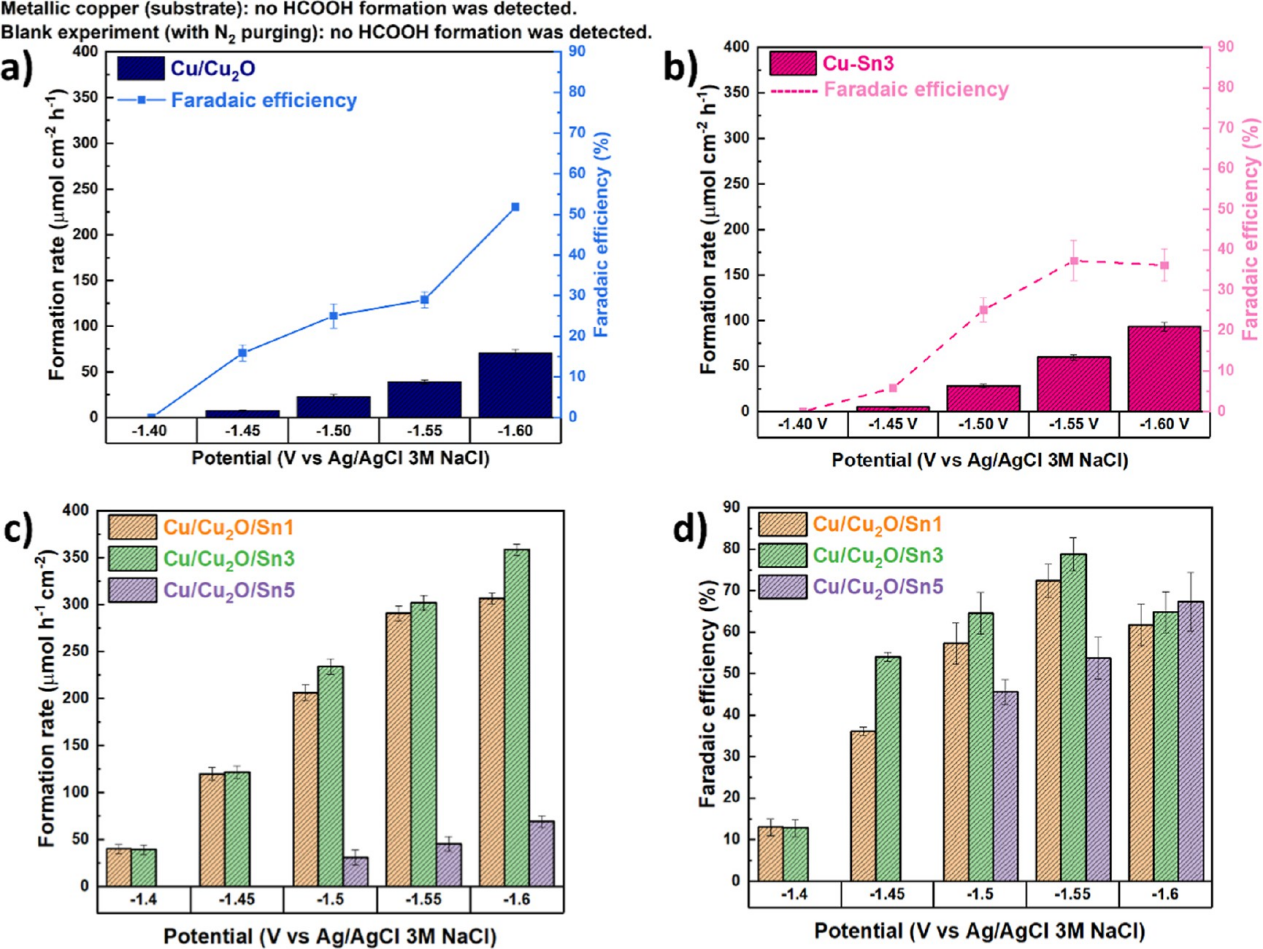
Production rate and faradaic efficiency of (a) Cu/Cu_2_O and (b) Cu–Sn3 electrodes for the electrochemical formation of HCOOH. (c) Production rate of HCOOH for Cu/Cu_2_O/Sn1, Cu/Cu_2_O/Sn3, and Cu/Cu_2_O/Sn5 electrodes. (d) Corresponding faradaic efficiencies of Cu/Cu_2_O/Sn1, Cu/Cu_2_O/Sn3, and Cu/Cu_2_O/Sn5 electrodes.

The electroreduction was also performed on the Cu/Sn alloy electrode (Cu–Sn3, Figure 7b), prepared with a 3 min Sn deposition—the same deposition time associated with optimal coverage for the Cu/Cu_2_O/Sn catalysts, i.e., the Sn deposition time that yielded the highest FE for HCOOH. without Cu_2_O, to isolate the effect of Cu_2_O deposition. Notably, Cu–Sn3 exhibited a low HCOOH formation rate and Faradaic efficiency, reaching 37% at −1.55 V, reinforcing once again the role of Cu_2_O in the formation rate and Faradaic efficiency (FE) toward HCOOH.

Upon Sn decoration, a substantial enhancement in performance was observed (Figure 7c,d). The HCOOH formation rates increased significantly for all Cu/Cu_2_O/Sn samples compared to Cu/Cu_2_O and the Cu–Sn alloy, reaching a value of 370 μmol cm^–2^ h^–1^ at −1.60 V. This improvement demonstrated the beneficial role of Sn in boosting CO_2_ reduction activity. Moreover, the Faradaic efficiencies (Figure 7d) displayed a remarkable increase, reaching 80% for Cu/Cu_2_O/Sn3 at −1.55 V, whereas Cu/Cu_2_O remained 30% at the same potential. The higher efficiencies indicated that Sn effectively suppressed the competing hydrogen evolution reaction (see Figure 10b), promoting CO_2_ reduction toward HCOOH as the dominant pathway. Comparing the different Sn loadings, Cu/Cu_2_O/Sn3 consistently achieved the best balance between activity and selectivity, showing both the highest formation rates and Faradaic efficiencies across the potential range. Lower loading (Sn1) enhanced activity but with slightly lower efficiency, while higher loading (Sn5) resulted in diminished rates and intermediate efficiencies, possibly due to excessive coverage of the Cu_2_O surface, which may have reduced the number of active sites or altered the surface electronic structure unfavorably. In summary, these results demonstrated that Sn decoration played a crucial role in steering the CO_2_ reduction pathway on Cu_2_O, significantly improving both HCOOH production rates and selectivity. The optimal Sn loading (Cu/Cu_2_O/Sn3) achieved the best catalytic performance, highlighting the importance of controlling the metal–oxide interface for efficient CO_2_ electroreduction.

The cycling experiments (Figure 8) revealed that the Cu/Cu_2_O/Sn-modified copper electrode exhibited both high activity and stability for the electrochemical reduction of CO_2_ to formic acid. In the first 1 h cycle, the amount of HCOOH formed was relatively lower (650 μmol), whereas from the second cycle onward, the production slightly increased (770 μmol) and remained nearly constant over subsequent cycles. This behavior was attributed to an initial activation of the catalytic surface, likely involving structural reorganization of the Cu/Cu_2_O/Sn domains under electrochemical conditions, which led to the exposure of additional active sites or stabilization of catalytically favorable phases.^67^ In parallel, the Faradaic efficiency remained consistently high, between 78–80% throughout all cycles, demonstrating that the selectivity toward HCOOH formation was preserved during repeated operation. These results indicated that, after a short activation stage, the catalyst achieved a stable performance regime.

**Figure 8:**
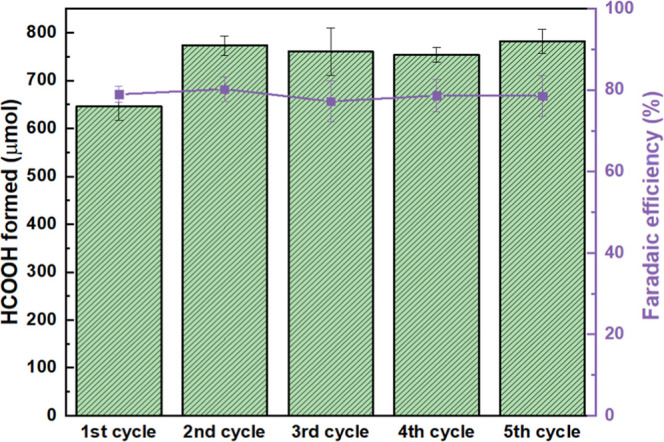
Amount of HCOOH formed (green bars, left axis) and corresponding faradaic efficiency (purple line, right axis) during consecutive electrochemical CO_2_ reduction cycles (60 min each) using Cu/Cu_2_O/Sn3 electrode.

The AFM images in Figure 9a–c displayed the surface morphology of the Sn-decorated Cu/Cu_2_O electrodes after electrochemical CO_2_ reduction. Compared to the as-prepared samples (Figure 4), no morphological modifications were observed, suggesting surface morphology stability of the catalysts under operating conditions.

**Figure 9:**
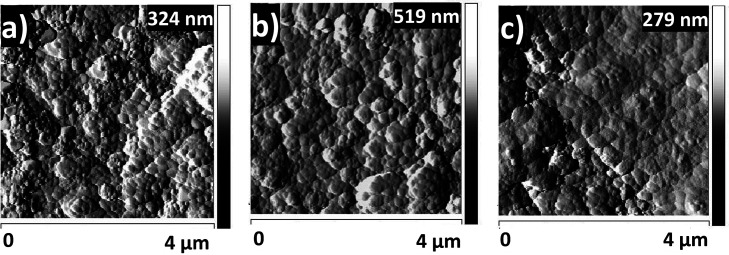
AFM topographic images of Sn-decorated Cu/Cu_2_O electrodes after CO_2_RR with different deposition times: (a) 1 min, (b) 3 min, and (c) 5 min.

For the samples decorated with Sn for 1, 3, and 5 min (Figure 9a–c), AFM analysis showed that the granular morphology remained essentially unchanged after electrolysis, with surface features and porosity similar to those of the corresponding annealed films used as starting materials. This indicates that electrochemical operation did not induce significant morphological changes, and the structural characteristics set by the Sn decoration and annealing were preserved throughout the reaction.

### 3.3 Flue Gas Electrocatalytic Conversion

Figure 10 presents the distribution of products obtained for CO_2_RR under pure CO_2_ and under plasma-derived products saturation, employed here as a simulated flue gas. All experiments were carried out under identical potential and flow conditions, enabling a direct comparison between the two scenarios.

**Figure 10:**
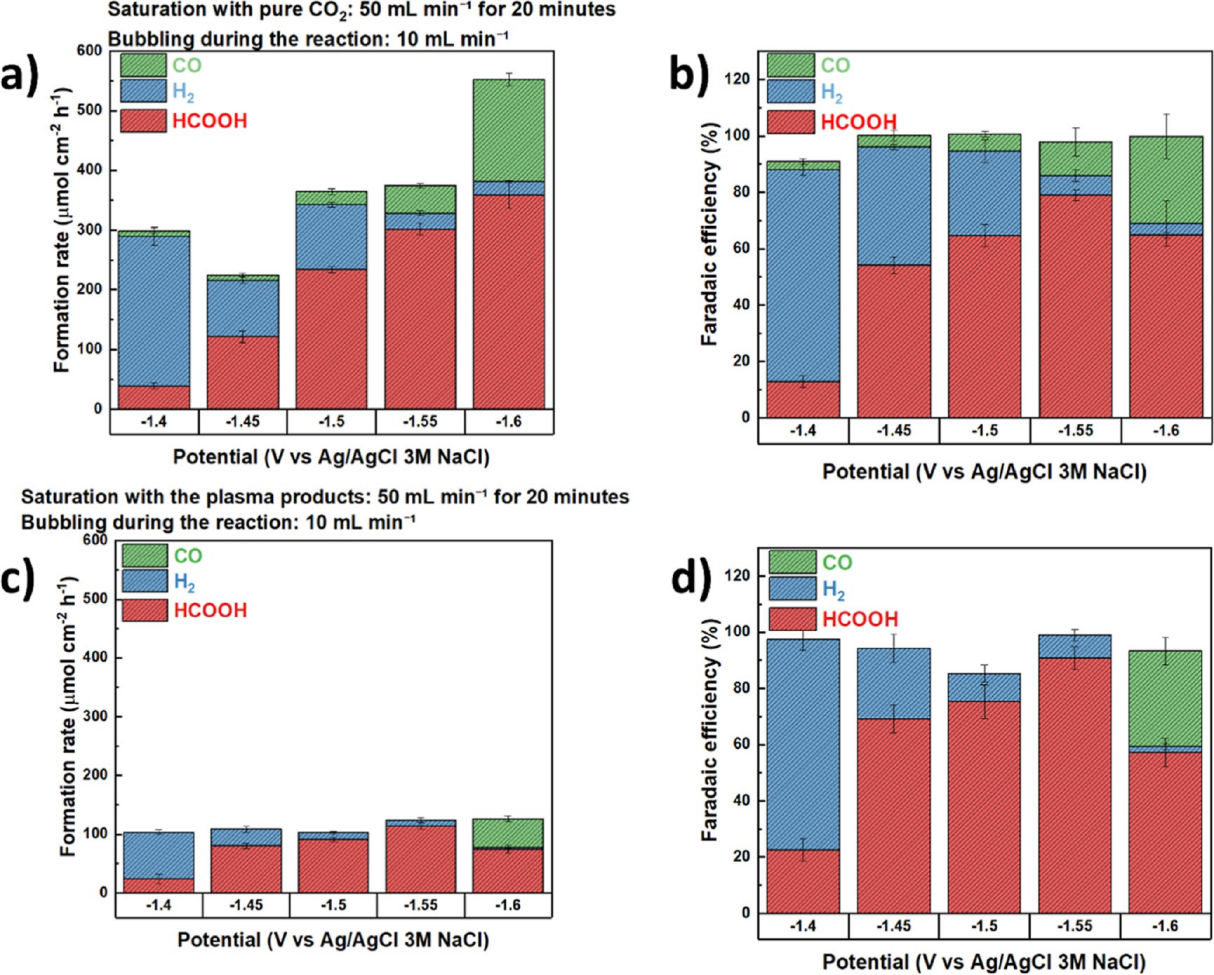
Product distribution from CO_2_RR at potentials between −1.40 and −1.60 V vs Ag/AgCl 3 M NaCl. (a) Formation rates and (b) Faradaic efficiencies under pure CO_2_. (c) Formation rates and (d) Faradaic efficiencies under plasma-derived gas (flue gas). The electrode employed in the experiments was the Cu/Cu_2_O/Sn3.

The quantitative analysis of reaction products (Figure 10a–d) further shows the impact of the feed composition. Under pure CO_2_ (Figure 10a,b), the HCOOH formation rate increased with potential, reaching 370 μmol cm^–2^ h^–1^ at −1.60 V, with Faradaic efficiencies up to 80% for Cu/Cu_2_O/Sn3 at −1.55 V. Minor amounts of CO and H_2_ were also detected, confirming that the incorporation of Sn enhanced the selectivity toward the formate pathway. In contrast, under the plasma-derived gas (Figure 10c,d), product formation rates were drastically reduced, remaining close to 100 μmol cm^–2^ h^–1^ across the potential range. Importantly, the product distribution observed with the plasma-derived gas differed markedly from the initial interpretation. Under the plasma-derived feed, H_2_ was the dominant product only at −1.40 V; at higher overpotentials, the selectivity shifted strongly in favor of formic acid. In particular, at −1.55 V, the FE for HCOOH reached 90%, exceeding the 80% FE measured for pure CO_2_ at the same potential, although the absolute HCOOH formation rate under the plasma-derived gas was substantially lower than that obtained with pure CO_2_. Thus, the plasma feed produced a trade-off: improved selectivity (higher FE) at certain potentials but reduced production rate (lower partial current to HCOOH). This indicated that under plasma-derived conditions the catalyst channeled a larger fraction of the total current toward formate, while overall kinetics were limited, most likely by reduced CO_2_ availability and by changes in the electrode surface/adsorbate environment induced by plasma products. In comparison to the state-of-the-art literature on electrochemical CO_2_ reduction using flue gas as a feedstock, our study demonstrated notable advancements. While previous research reported Faradaic efficiencies (FEs) for formate production ranging from 39% to 90% under flue gas conditions,^12,16,68^ our Cu/Cu_2_O/Sn3 catalyst achieved a FE of approximately 90% at −1.55 V. This enhancement in selectivity is attributed to the incorporation of tin, which modulates the catalyst surface to favor the formate pathway. Although Van Daele et al. (2024)^12^ also reported Faradaic efficiencies (FEs) of 90% for formate production under flue gas conditions containing SO_2_ and NO, their system required careful management of O_2_ impurities, as the presence of 1% O_2_ led to a significant decrease in FE due to competing oxygen reduction reactions. In contrast, the Cu/Cu_2_O/Sn3 catalyst developed in this work achieved an FE of 90% at −1.55 V under plasma-derived gas conditions without additional measures to mitigate O_2_ (5% O_2_). While the simulated flue gas experiments included NO_*x*_ species, SO_2_ was not considered in the present study. Sulfur containing compounds are well-known catalyst poisons for Cu based electrodes, as they can strongly adsorb and form stable metal–sulfur species, leading to site blocking and activity loss. Previous studies indicate that Sn and SnO_*x*_ modified surfaces exhibit a reduced affinity for sulfur compared to bare Cu, and that Cu–Sn interfaces may partially mitigate sulfur poisoning by altering the electronic structure and adsorption energetics of sulfur species.^69^ Nevertheless, prolonged exposure to SO_2_ is still expected to negatively impact catalytic performance.

In the case of pure CO_2_, the LSV and CV curves (Figures 2 and 5) showed a pronounced increase in cathodic current at more negative potentials, indicating the occurrence of CO_2_ reduction. In particular, the Cu/Cu_2_O/Sn3 electrode displayed the highest activity, with a clear distinction between curves collected in N_2_ and in CO_2_, evidencing that additional currents were directly associated with CO_2_ reduction intermediates. Under the plasma-derived gas, the voltammetric response differed systematically from that observed with pure CO_2_: the onset of cathodic activity was shifted to more negative potentials, indicating a higher overpotential requirement for electrochemical activation (see Figure 11). This shift implied altered kinetics and/or diminished availability of reducible CO_2_ at the electrode surface. Importantly, the plasma feed did not simply produce uniformly lower currents across all potentials. At the lowest applied potential (−1.40 V), H_2_ was the predominant product, whereas at more negative potentials (notably −1.55 V) the electrode channeled a substantially larger fraction of the current to formic acid.

**Figure 11:**
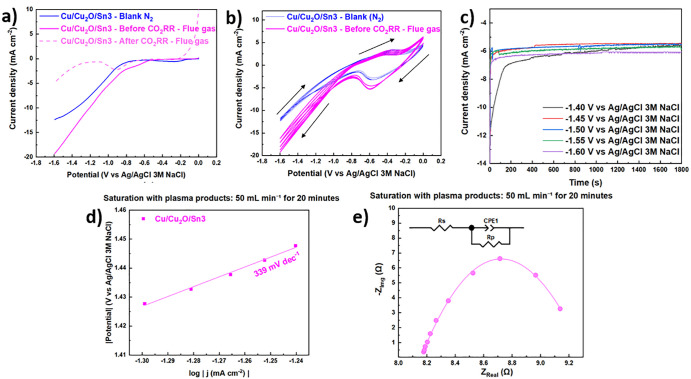
(a) Linear sweep voltammetry (LSV, scan rate 5 mV s^–1^), (b) cyclic voltammetry (CV, 10 consecutive cycles, scan rate 50 mV s^–1^), (c) chronoamperometric curves of Cu/Cu_2_O/Sn3 electrode, (d) Tafel plots derived from the corresponding LSV curve and (e) Nyquist plot and fitted curve obtained from electrochemical impedance spectroscopy (EIS) measurements for the Cu/Cu_2_O/Sn3 catalyst. Measurements under simulated plasma-derived flue gas for the CO_2_RR. All experiments were carried out in a two-compartment electrochemical cell using 0.2 M KHCO_3_ as the catholyte and 1 M KOH as the anolyte.

When the Cu/Cu_2_O/Sn3 catalyst was evaluated under the plasma-treated flue gas stream, clear kinetic differences emerged relative to the measurements performed in pure CO_2_. The Tafel slope increased to 339 mV dec^–1^ (Figure 11d), far higher than the 73 mV dec^–1^ obtained under pure CO_2_. This steep slope indicates that the rate-determining step becomes significantly slower in the presence of the plasma-derived flue gas, likely due to competitive adsorption between CO_2_-derived intermediates and the additional species present in the flue gas, or to subtle surface modifications induced by the plasma process.

EIS measurements further illuminate how the altered gas composition affects interfacial processes. The solution resistance remained similar (*R*_s_ = 8.178 Ω), confirming that the electrolyte environment did not change appreciably. Interestingly, the charge-transfer resistance decreased to 1.169 Ω, a slightly lower value than that measured under pure CO_2_ (1.637 Ω). This suggests that the plasma generated species may facilitate electron transfer at the electrode surface. At the same time, the CPE-T parameter increased to 0.023, indicating a noticeable rise in interfacial capacitance, consistent with a more heterogeneous or dynamically reconstructed surface under flue gas exposure. Despite the lower *R*_p_, the larger Tafel slope demonstrates that the intrinsic catalytic pathway becomes kinetically limited under these conditions. Thus, while electron transfer to the surface may be slightly favored, the subsequent steps involving adsorption, activation, or stabilization of intermediates become significantly less efficient. Overall, the plasma-generated flue gas introduces new kinetic constraints that hinder CO_2_ reduction relative to pure CO_2_, highlighting the sensitivity of Cu_2_O/Sn interfaces to gas phase composition and surface adsorbate interactions.

The stability tests (Figure 12) highlighted the robustness of the system under different feed gases. With pure CO_2_, the system exhibited an initial activation step followed by stable performance, producing 770 μmol of HCOOH after the second cycle, with Faradaic efficiencies of 78–80% maintained over five consecutive cycles. When operating under plasma-derived flue gas, HCOOH production remained consistently high, with Faradaic efficiencies of approximately 90% throughout all cycles.

**Figure 12:**
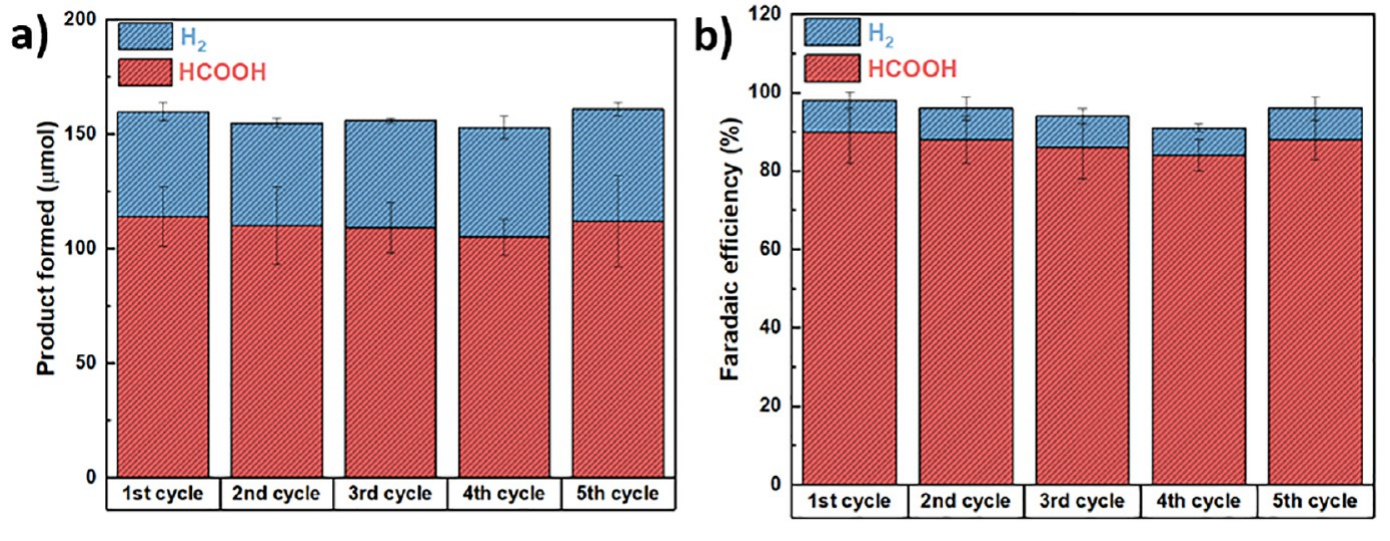
Amount of HCOOH formed (a) and corresponding faradaic efficiency (b) during consecutive electrochemical CO_2_ reduction cycles (60 min each) using Cu/Cu_2_O/Sn3 electrode and plasma-derived flue gas, employing 0.2 M KHCO_3_ as the catholyte and 1 M KOH as the anolyte. The applied potential was −1.55 V vs Ag/AgCl (3 M NaCl).

The direct comparison between both feed conditions highlighted three key aspects: (1) under pure CO_2_, the Cu/Cu_2_O/Sn3 electrode provided high activity and selectivity toward HCOOH, with interfacial Cu–Sn sites playing a crucial role in stabilizing formate intermediates; (2) under the plasma-derived gas, the activity were strongly suppressed due to the limited CO_2_ availability but we still obtained good selectivity for HCOOH; and (3) these results demonstrated that although the catalyst performed efficiently under ideal CO_2_ conditions, its practical application in real flue gas streams required additional strategies, such as CO_2_ enrichment and optimization of mass transport. In summary, while experiments with pure CO_2_ demonstrated the intrinsic efficiency of Cu/Cu_2_O/Sn3 for selective HCOOH production, those with plasma-derived gas revealed the practical challenges of operating under more realistic flue gas conditions, where the limited CO_2_ concentration compromised electrochemical conversion.

The XPS spectra collected after the electrochemical tests under flue gas (Figure 13) reveal additional surface changes that reflect the chemical complexity introduced by the reactive gas mixture. The most evident difference is the emergence of a clear N 1s peak, which was not observed previously. This signal confirms the adsorption of nitrogen-containing species, most likely nitrates or nitrites formed from NOx present in the flue gas. Its appearance, together with the increased intensity of the higher-binding-energy component in the O 1s region (∼532 eV), indicates that nitrogen- and oxygen-rich adsorbates accumulated on the surface during reaction. These species likely coexist with surface hydroxyls, leading to a more heterogeneous oxygen environment compared to the smoother O 1s profile seen in the postannealed sample.^49^ The Cu 2p region shows that the potential-variation sample exhibits higher peak intensity, while the cycled sample displays broader peaks, indicating increased surface heterogeneity and partial reoxidation. Similarly, in the Sn 3d region, the cycled electrode presents slightly broader and shifted peaks compared to the potential-variation sample, consistent with surface restructuring and reversible redox changes.

**Figure 13:**
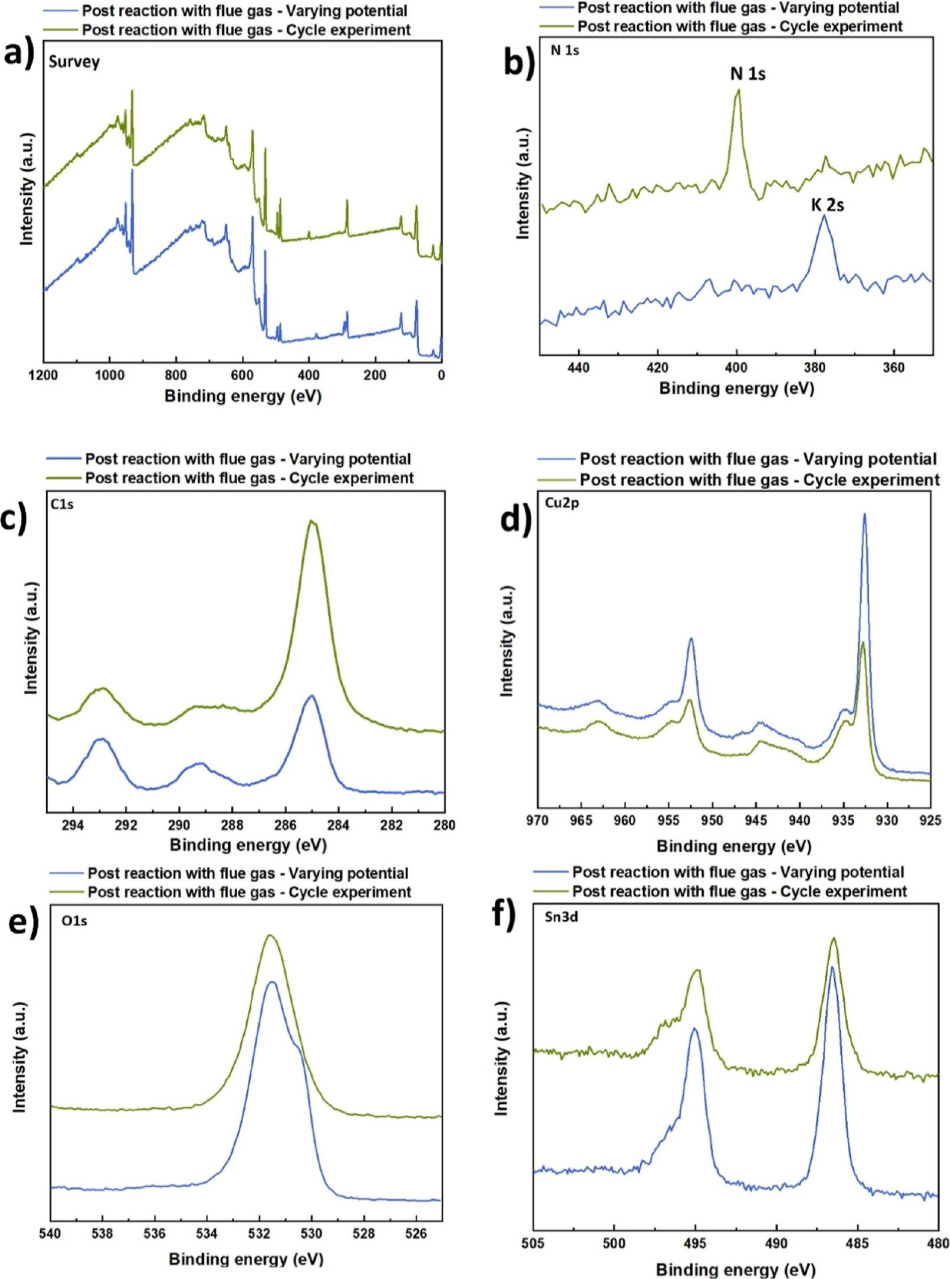
XPS spectra after CO_2_RR bubbling plasma products flue gas: (a) survey spectra showing the main elements present in the samples, (b) N 1s spectra, (c) C 1s spectra, (d) Cu 2p spectra, (e) O 1s spectra, (f) Sn 3d spectra.

The Cu/Cu_2_O/Sn3 sample tested under varying potential exhibited a copper content of 26.4 at %, oxygen at 71.2 at %, and only a minor amount of tin (2.4 at %). Similarly, the sample subjected to cycling displayed 23.8 at % Cu, 74.1 at % O, and 2.0 at % Sn. Compared to the pristine Cu_2_O and the Sn-deposited and annealed samples, these flue gas-exposed samples show a markedly lower tin content, suggesting that most of the surface tin was either leached or redistributed during electrochemical testing under realistic conditions. Additionally, the increase in copper relative to oxygen indicates a partial reduction of the oxide surface, which could be attributed to the applied potential and repeated cycling. Overall, when comparing the surface composition of these flue gas-exposed samples with the Sn-deposited samples it becomes clear that the combination of potential variation and cycling under flue gas conditions significantly alters the surface chemistry. While the deposition and annealing steps effectively introduced and stabilized tin at the surface, exposure to electrochemical operation in flue gas leads to a depletion of tin and subtle changes in the Cu/O ratio, highlighting the dynamic nature of the Cu–Sn interface under operating conditions.

## 4 Conclusions

This study shows that controlled Sn decoration of Cu/Cu_2_O electrodes, combined with annealing and electrochemical activation, allows fine adjustment of their catalytic behavior toward CO_2_ electroreduction. The deposition time proved to be a crucial parameter, as it determined both the degree of Sn coverage and the formation of interfacial Sn–Cu domains. A 3 min deposition provided the best compromise between activity and selectivity for HCOOH production. Annealing at 500 °C induced grain coarsening, Sn diffusion, and partial formation of Cu–Sn intermetallic phases, which further modified the surface structure and catalytic response. Under pure CO_2_, the Cu/Cu_2_O/Sn3 electrode reached Faradaic efficiencies of up to 80% and exhibited high HCOOH production rates, while longer deposition times (5 min) led to excessive Sn coverage, suppressing Cu active sites and reducing selectivity. When tested under plasma-derived acid gas-containing flue gas, the system revealed a clear trade-off. Namely, the overall formation rate decreased due to the lower CO_2_ partial pressure in the feed, but selectivity toward HCOOH increased, reaching up to 90% FE. Raman and AFM analyses showed extensive surface reorganization under these conditions, involving carbonate formation, dissolution–redeposition, and heterogeneous surface roughening. Together, these results emphasize the decisive role of interfacial structure and surface dynamics in governing both catalytic activity and durability. More broadly, they illustrate the challenges and opportunities associated with oxide-derived Cu–Sn catalysts for CO_2_ conversion in realistic flue gas environments, where high selectivity can be maintained but productivity remains constrained by feed composition.
